# PVR (CD155) Expression as a Potential Prognostic Marker in Multiple Myeloma

**DOI:** 10.3390/biomedicines10051099

**Published:** 2022-05-10

**Authors:** Byung-Hyun Lee, Ji-Hea Kim, Ka-Won Kang, Se-Ryeon Lee, Yong Park, Hwa-Jung Sung, Byung-Soo Kim

**Affiliations:** 1 Department of Internal Medicine, Korea University College of Medicine, Seoul 02841, Korea; potato0430@hanmail.net (B.-H.L.); ggm1018@gmail.com (K.-W.K.); logost@hanmail.net (S.-R.L.); paark76@hanmail.net (Y.P.); 2 Department of Biomedical Science, Graduate School of Medicine, Korea University, Seoul 02841, Korea; redmongi@nate.com

**Keywords:** multiple myeloma, PVR, CD155, TIGIT, plasma cells, prognosis

## Abstract

Poliovirus receptor (PVR, CD155) is upregulated during tumor progression, and PVR expression is associated with poor prognosis in cancer patients; however, prognostic implications for PVR in multiple myeloma (MM) have not been investigated. PVR plays an immunomodulatory role by interacting with CD226, CD96, and TIGIT. TIGIT is a checkpoint inhibitory receptor that can limit adaptive and innate immunity, and it binds to PVR with the highest affinity. We used immunohistochemistry, ELISA, qPCR, and flow cytometry to investigate the role of PVR in MM. PVR was highly expressed in patients with MM, and membrane PVR expression showed a significant correlation with soluble PVR levels. PVR expression was significantly associated with the Revised-International Staging System stage, presence of extramedullary plasmacytoma and bone lesion, percentage of bone marrow plasma cells (BMPCs), and β2-microglobulin levels, suggesting a possible role in advanced stages and metastasis. Furthermore, TIGIT expression was significantly correlated with the percentage of BMPCs. Patients with high PVR expression had significantly shorter overall and progression-free survival, and PVR expression was identified as an independent prognostic factor for poor MM survival. These findings indicate that PVR expression is associated with MM stage and poor prognosis, and is a potential prognostic marker for MM.

## 1. Introduction

The poliovirus receptor (PVR, CD155), a member of the nectin-like protein family, has recently emerged as a promising target for immunotherapy to enhance antitumor responses [1]. PVR is upregulated during tumor progression and promotes tumor proliferation and migration [2]. Additionally, PVR present on the cancer cell surface reportedly promotes tumor invasiveness, and its upregulation in tumor-infiltrating myeloid cells impairs antitumor T lymphocyte and natural killer (NK) cell functions, thereby suppressing antitumor immunity [3]. Moreover, this upregulation is associated with an increased metastatic capacity of cancer cells [4]. Furthermore, PVR is overexpressed in several types of cancers, including serous ovarian cancer, colorectal carcinoma, hepatocellular carcinoma, lung adenocarcinoma, esophageal small cell carcinoma, and cholangiocarcinoma [5,6,7,8,9,10], and highly expressed in bone marrow cells in multiple myeloma (MM) [11]. Thus, the effects of PVR expression on progression and metastasis in patients with MM are of interest.

There is an increasing interest in the prognostic effects of PVR expression in patients with cancer, as an elevated PVR expression is associated with poor survival in most cancer types [8,9,10,12]. For instance, in a previous study conducted on patients with lung cancer, PVR-positive patients had significantly shorter overall survival (OS) and progression-free survival (PFS) than PVR-negative patients [8]. In another study, esophageal cancer patients in the PVR-positive group showed unfavorable survival compared to those in the PVR-negative group [9]. Additionally, PVR expression is an independent poor prognostic factor for OS in patients with cholangiocarcinoma [10], and high *PVR* mRNA levels are associated with shorter OS and a recurrence-free interval in patients with breast cancer [12]. In a study of hematologic malignancies, high PVR expression was identified as an independent negative prognostic marker for survival in patients with acute myeloid leukemia (AML) [13]. However, the clinical and prognostic significance of PVR in patients with MM has not yet been reported, thereby requiring further studies to determine the prognostic value of PVR expression in MM.

T cell immunoreceptors with Ig and ITIM domains (TIGIT) is a class of transmembrane glycoprotein expressed on NK cells and T cell subsets [14]. TIGIT expression is upregulated in both T and NK cells, where it inhibits cytotoxic activity [15]. TIGIT has several ligands, including PVR, CD112, and CD113, with PVR interactions showing the highest affinity [14]. PVR plays an immunomodulatory role by interacting with TIGIT, CD226, and CD96, and TIGIT binds PVR with the highest affinity, followed by CD96 and CD226 [14]. TIGIT expression appears to be associated with advanced disease status and poor clinical outcomes in several cancers [16,17,18]. A previous meta-analysis of cancers demonstrated that a high TIGIT expression is correlated with worse OS and PFS [19], and in hematological malignancies, high TIGIT expression is associated with poor clinical outcomes in patients with follicular lymphoma [20] and AML [21,22]. In a previous study on MM, TIGIT expression was upregulated in CD8^+^ T cells during myeloma progression and associated with impaired effector functions [23]. However, the prognostic implication of TIGIT expression in patients with MM is still unclear.

Therefore, in this study, we evaluate PVR expression and investigate whether its expression has a prognostic role in patients with MM. Additionally, we evaluated the relationship of TIGIT expression, as the primary PVR ligand, with clinical factors. Furthermore, we assessed PVR as a potential immune biomarker for predicting the prognosis of patients with newly diagnosed MM.

## 2. Materials and Methods

### 2.1. Patients

We evaluated patients with newly diagnosed MM who underwent a bone marrow examination at the Korea University Anam Hospital (Seoul, Korea). This retrospective cohort included 171 patients diagnosed with MM between May 2010 and October 2019. Of these, 46 were excluded because their bone marrow specimens were unavailable. Thus, this study examined 125 bone marrow specimens obtained at the time of diagnosis. Additionally, bone marrow aspiration samples were collected from 22 patients with multiple myeloma for validation, four monoclonal gammopathy of undetermined significance (MGUS) patients, and three patients without cancer for comparative purposes between July 2018 and October 2019. The International Myeloma Working Group criteria was used for the diagnosis of MM [24]. The study was conducted according to the guidelines of the Declaration of Helsinki and approved by the Institutional Review Board of the Korea University Medical Center (No. 2018AN0150). The patients provided written informed consent to participate in the study.

### 2.2. Immunohistochemistry (IHC) Staining and Analysis

Formalin-fixed and paraffin-embedded bone marrow blocks were cut into 4–5 µm sections and baked at 60 °C for 1 h in an incubator. The sections were deparaffinized with xylene and hydrated through graded ethanol into water. Antigen retrieval was performed in sodium EDTA buffer (pH 9.0) by heating the sections using a pressure cooker. The sections were incubated with 3% hydrogen peroxide for 10 min to block endogenous peroxidase activity. The slides were incubated with primary antibodies at 4 °C overnight. The following primary antibodies were used: rabbit CD155 (PVR) polyclonal antibody (1:100; Elabscience, Houston, TX, USA) and mouse CD138 monoclonal antibody (clone B-A38; 1:100; Cell Marque, Rocklin, CA, USA) to double stain by using the Polink DS-MR-Hu C1 kit (GBI Labs, Bothell, WA, USA) according to manufacturer instructions. Optical microscopy was used for image acquisition and examination (DS-Fi2; Nikon Metrology, Tokyo, Japan). The proportion score of PVR was grouped into four categories according to the percentage of stained PVR expressing plasma cells (0, <1%; 1, 1–10%; 2, 10–50%; and 3, >50%). The staining intensity of PVR was measured based on the average intensity of the positively stained plasma cells and divided into four categories (0, none; 1, weak; 2, intermediate; and 3, strong). A histoscore (H-score) was calculated by adding proportion score values to staining intensity score, which ranged from 0 to 6. The optimal cut-off value for high PVR expression was determined by the Contal and O’Quigley’s method, which was based on the absolute value of the maximal log-rank statistic [25]. H-scores were manually evaluated by three independent investigators and interobserver variability was tested using intraclass correlation coefficient (ICC). The consistency was assessed as being excellent based on the value of an ICC (0.973; *p* < 0.001).

### 2.3. Gene-Expression Analysis

Total RNA was extracted using TRIzol reagent (Invitrogen, Carlsbad, CA, USA) according to the manufacturer’s instructions. Complementary DNA (cDNA) was synthesized from 1 μg of total RNA using the LaboPass cDNA synthesis kit (Cosmogenetech, Seoul, Korea). The quantitative real-time polymerase chain reaction (qPCR) was performed on a CFX96 Touch real-time PCR detection system (Bio-Rad, Hercules, CA, USA) using Labopass SYBR Green Q master mix (Cosmogenetech) according to the manufacturer’s protocol. The primers used were as follows: *PVR*, 5′-CTG GCT CCG AGT GCT TGC-3′ and 5′-GAG GTT CAC AGT CAG CA-3′. *Glyceraldehyde 3-phosphate dehydrogenase* was used as the internal control. Relative *PVR* transcript levels were determined using the 2^−ΔΔCt^ method, with all samples tested in triplicate and mean values used for further analyses. 

### 2.4. Enzyme-Linked Immunosorbent Assay (ELISA)

Bone marrow cell lysates were prepared using the freeze (−20 °C)-thaw (room temperature) method. PVR protein in each cell lysate was quantified using a human PVR/CD155 ELISA kit (LS-F65983; LSBio, Seattle, WA, USA) according to manufacturer instructions, and soluble PVR and TIGIT proteins were measured in bone marrow plasma using a different human PVR/CD155 ELISA kit (MBS1752576; MyBioSource, San Diego, CA, USA) and a human TIGIT ELISA kit (MBS1607096; MyBioSource), respectively, according to manufacturer instructions. All samples were tested in duplicate, and the mean values were used for further analyses.

### 2.5. Flow Cytometry 

Bone marrow mononuclear cells were incubated with fluorescence-dye-conjugated antibodies for 15 min at 4 °C. Antibodies used for staining were CD3 (clone SK7; BD Biosciences, Franklin Lakes, NJ, USA), CD8 (clone RPA-T8; BD Biosciences), CD56 (clone B159; BD Biosciences), and TIGIT (clone A15153G; BioLegend, San Diego, CA, USA). Stained cells were evaluated by flow cytometry using a FACSVerse instrument (BD Biosciences), and the obtained data were analyzed using the FlowJo software (Tree Star Inc., Ashland, OR, USA).

### 2.6. Statistical Analysis

Categorical variables were evaluated using the chi-squared or Fisher’s exact test, and continuous variables were evaluated using Student’s *t*-test or Mann–Whitney *U* test. The Kruskal–Wallis test was used to compare three unmatched groups, and Dunn’s multiple-comparison test was used for post-hoc analysis. Pearson’s correlation coefficient was used to determine the relationship between the two variables. OS was measured from the time of diagnosis to death from any cause. PFS was defined as the time from the start of treatment to disease progression or death from any cause. The Kaplan–Meier survival curve was used to analyze time to event data, and the log-rank test was used to assess differences in survival curves between patient groups. Cox proportional hazards model was used to assess the multivariable relationships between prognostic factors and survival outcomes, and we observed no major violation of the proportional hazards assumption when using the Schoenfeld method. All tests were two-sided, and a *p* < 0.05 was considered significant. Statistical analyses were performed using GraphPad Prism (v9.2.0; GraphPad Software, La Jolla, CA, USA) and SPSS (v25.0; IBM Corp, Armonk, NY, USA).

## 3. Results

### 3.1. PVR and TIGIT Expression Relative to Clinical Factors

**Figure 1:**
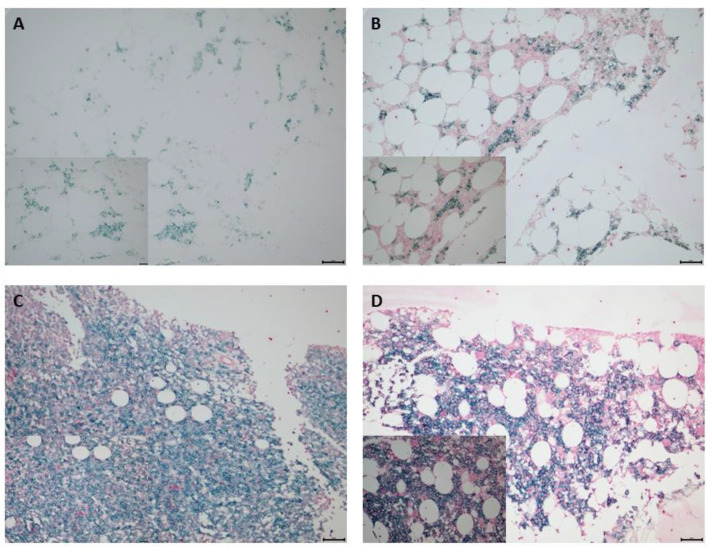
IHC staining to determine PVR expression in bone marrow plasma cells obtained from patients with MM. CD138^+^ plasma cells were stained using Emerald chromogen (blue-green color) and PVR-expressing cells were stained using Permanent Red chromogen (red color). Representative images depicting low and high PVR expression based on H-score. H-scores of (**A**) 0 and (**B**) 2 represented low PVR expression, and (**C**) 4 and (**D**) 6 were used to represent high PVR expression. Top row, original magnification: 200×; bottom row (inset), original magnification: 400×. H-score, histoscore; IHC, immunohistochemistry; MM, multiple myeloma; PVR, poliovirus receptor.

**Figure 2:**
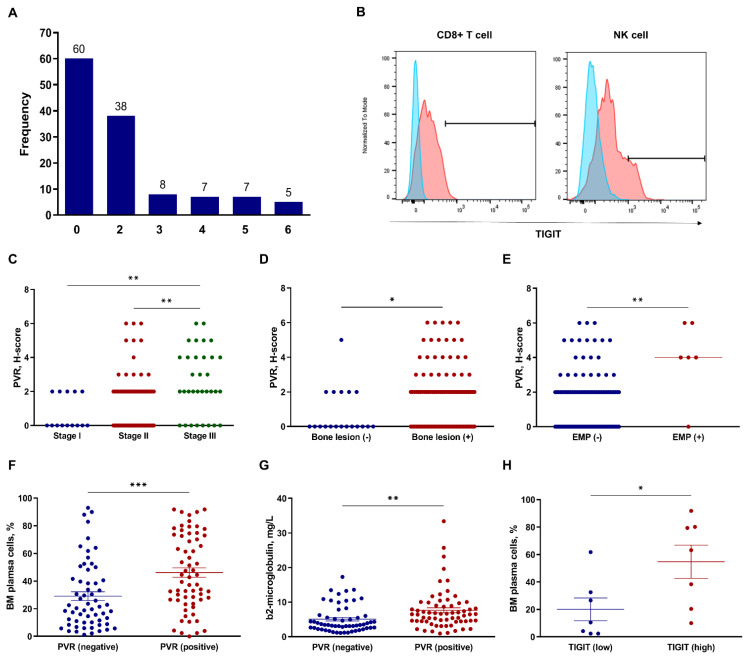
Assessment of PVR and TIGIT expression in relation to clinical factors in patients with MM. (**A**) Distribution of PVR expression (H-score) in bone marrow plasma cells in all patients. (**B**) Representative histogram of TIGIT expression in CD8^+^ T cells and NK cells. Comparison of PVR expression based on (**C**) R-ISS, presence of (**D**) bone lesion, and (**E**) EMP. * *p* < 0.05, ** *p* < 0.01; Kruskal–Wallis and Mann–Whitney *U* tests. Comparison of clinical factors based on PVR- and TIGIT-expression status. (**F**) Percentage of bone marrow plasma cells and (**G**) concentration of β2-microglobulin in the negative (H-score: 0) and positive (H-score: > 0) PVR-expression groups (n = 125). Error bars indicate the mean ± standard error of the mean. ** *p* < 0.01, *** *p* < 0.001; unpaired *t*-test. (**H**) Percentage of bone marrow plasma cells in the low (<median) and high (≥median) TIGIT-expression groups (n = 14). * *p* < 0.05; Mann–Whitney *U* test. BM, bone marrow; EMP, extramedullary plasmacytoma; H-score, histoscore; MM, multiple myeloma; NK, natural killer; PVR, poliovirus receptor; R-ISS, Revised International Staging System; TIGIT, T cell immunoreceptor with Ig and ITIM domains.

We investigated 125 bone marrow biopsy samples for expression of PVR in plasma cells and clinical parameters, with PVR expression examined via double-staining IHC. Figure 1 shows the representative images based on the H-score, and Figure 2A shows the distribution of PVR expression in patients with MM. We then examined the 14 available bone marrow samples to assess TIGIT expression in CD8^+^ T and NK cells using flow cytometric analysis (Figure 2B), with the original flow cytometric data shown in Table S1.

We then compared PVR and TIGIT expression between categorical clinical parameters, including age, Eastern Cooperative Oncology Group (ECOG) performance status (<2 vs. ≥2), serum free-light chain ratio [high (≤0.01 or ≥100) vs. low], cytogenetic abnormalities (high risk vs. others), Revised International Staging System (R-ISS) stage (I vs. II vs. III), osteolytic bone lesion (presence vs. absence), and extramedullary plasmacytoma (EMP) (presence vs. absence). High-risk cytogenetics were defined as t(4;14), t(14;16), del(17/17p), *TP53* deletion, or chromosome 1 abnormalities, including gain(1q) and del(1p). Patients with R-ISS stage III showed significantly higher PVR-expression levels than those with stage I (*p* = 0.004) and II (*p* = 0.001) (Figure 2C). Additionally, patients with bone lesion (*p* = 0.034) (Figure 2D) and EMP (*p* = 0.002) (Figure 2E) showed significantly higher PVR expression than those without bone lesion and EMP. Patients with positive PVR expression (H-score > 0) showed significantly higher C-telopeptide of type I collagen (CTX) levels, a bone resorption marker (*p* = 0.032; Figure S1a), than patients with negative PVR expression (H-score = 0); however, no significant difference was observed between PVR expression and osteocalcin levels, a bone formation marker (*p* = 0.891; Figure S1b). Analysis of continuous clinical parameters based on PVR- and TIGIT-expression status included age, serum M-protein levels, percentage of bone marrow plasma cells, β2-microglobulin levels, and lactate dehydrogenase (LDH) levels. Patients with positive PVR expression showed significantly higher bone marrow plasma cell (mean, 29.10% vs. 46.15%; *p* < 0.001) (Figure 2F) and β2-microglobulin (mean, 5.064 mg/L vs. 7.642 mg/L; *p* = 0.005) (Figure 2G) levels than patients with negative PVR expression. Moreover, patients with high TIGIT expression in CD8^+^ T cells (cut-off point, median) showed a significantly higher percentage of bone marrow plasma cells (*p* = 0.037) (Figure 2H) than patients with low TIGIT expression. No significant differences were found among other factors.

### 3.2. Analysis of PVR Expression Using Different Detection Methods

**Figure 3:**
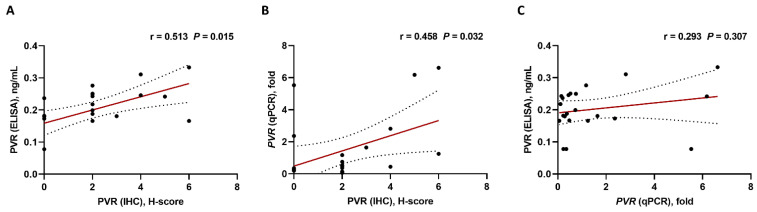
Correlations based on PVR expression using different detection methods (n = 22). (**A**) IHC vs. ELISA, (**B**) IHC vs. qPCR, and (**C**) qPCR vs. ELISA. Pearson’s or Spearman’s correlation coefficients (r) were used for analyses. Black ball, raw data; red line, linear fit line; dotted line, 95% confidence band. ELISA, enzyme-linked immunosorbent assay; IHC, immunohistochemistry; qPCR, quantitative polymerase chain reaction.

For validation of the results of PVR-expression analysis by the H-score, PVR protein levels were analyzed using ELISA and *PVR* mRNA levels using qPCR in the available 22 bone marrow aspiration specimens (Table 1). The PVR protein levels measured via ELISA showed a significant correlation with the H-score from IHC analysis (r = 0.513, *p* = 0.015) (Figure 3A). Moreover, the *PVR* mRNA levels evaluated using qPCR showed a significant correlation with PVR protein levels determined by the H-score from IHC analysis (r = 0.458, *p* = 0.032) (Figure 3B); however, we observed no significant correlations between *PVR* mRNA levels and the protein levels determined by ELISA (r = 0.293, *p* = 0.307) (Figure 3C). 

### 3.3. Analysis of Soluble PVR and TIGIT Levels

**Figure 4:**
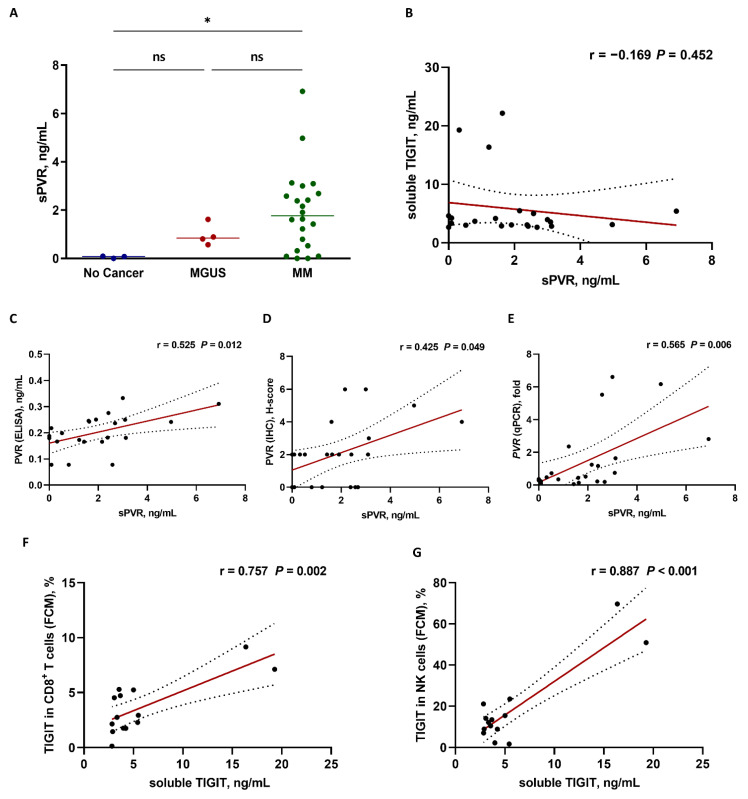
Correlation analysis based on soluble PVR (sPVR) and TIGIT levels in bone marrow plasma from patients with MM. (**A**) sPVR levels from patients without cancer (n = 3), patients with MGUS, (n = 4), and patients with MM (n = 22). * *p* < 0.05, ns, not significant; Kruskal–Wallis test. (**B**) Relationship between sPVR and soluble TIGIT (n = 22). No significant correlations were observed. Positive correlation between sPVR and PVR expression in bone marrow plasma cells as measured by (**C**) ELISA, (**D**) IHC, and (**E**) qPCR (n = 22). Positive correlation between soluble TIGIT and TIGIT expression in bone marrow (**F**) CD8^+^ T cells and (**G**) NK cells (n = 14) measured by FCM. Pearson’s or Spearman’s correlation coefficients (r) were used for analyses. Black ball, raw data; red line, linear fit line; dotted line, 95% confidence band. BM, bone marrow; ELISA, enzyme-linked immunosorbent assay; FCM, flow cytometry; IHC, immunohistochemistry; MGUS, monoclonal gammopathy of undetermined significance; MM, multiple myeloma; PVR, poliovirus receptor; qPCR, quantitative polymerase chain reaction; TIGIT, T cell immunoreceptor with Ig and ITIM domains.

Because PVR and TIGIT proteins can be found in soluble forms, we quantified soluble PVR protein levels in bone marrow plasma from patients with MM, MGUS, and patients without cancer. Although we found soluble PVR in both MGUS and patients with MM, we detected no significant differences in levels between the two patient subsets, although soluble PVR levels were significantly higher in patients with MM relative to those in patients without cancer (*p* = 0.042) (Figure 4A). We identified soluble TIGIT in patients with MM but no significant correlations between soluble TIGIT and PVR protein levels (Figure 4B). 

We then examined the relationship between soluble PVR levels and surface PVR expression using various detection methods. Soluble PVR levels were significantly correlated with the surface PVR expression data obtained using ELISA (r = 0.525, *p* = 0.012) (Figure 4C), IHC (r = 0.425, *p* = 0.049) (Figure 4D), as well as mRNA levels obtained with qPCR (r = 0.565, *p* = 0.006) (Figure 4E). We then examined the relationship between soluble TIGIT level and surface TIGIT-expression levels. Soluble TIGIT levels also positively correlated with surface TIGIT expression in CD8^+^ T cells (r = 0.757, *p* = 0.002) (Figure 4F) and NK cells (r = 0.887, *p* < 0.001) (Figure 4G). ELISA data for soluble PVR and TIGIT protein levels are shown in Table S2.

### 3.4. Patient Characteristics According to PVR-Expression Status

We determined the optimal cut-point value for high or low PVR expression based on the H-score of 3. Based on an H-score ≥ 3, we classified 27 patients as having high PVR expression and 98 as having low PVR expression. Table 2 summarizes patient characteristics according to PVR-expression status. We found no significant difference in median age between the high PVR expression [66.0; interquartile range (IQR): 58.0–72.0 years] and low PVR expression (66.0; IQR, 58.0–72.3 years) groups (*p* = 0.865). In total, 16 patients (59.3%) with high PVR expression and 37 (37.8%) with low PVR expression showed high LDH levels (greater than the upper limit of normal [ULN]; *p* = 0.045). Thirteen (48.1%) and 28 (28.6%) patients in the high and low PVR groups, respectively, had high-risk cytogenetic abnormalities (*p* = 0.055), and patients in the high PVR group showed a higher percentage of bone marrow plasma cells (46.5; IQR, 25.9–77.0) compared with those in the low PVR group (30.4; IQR, 5.39–10.5) (*p* = 0.018). Additionally, patients in high PVR group had high β2-microglobulin levels (6.63; IQR, 25.9–77.0 mg/L) compared with patients in low PVR group (4.44; IQR, 2.66–7.87 mg/L) (*p* = 0.004), and hemoglobin levels were lower in the high PVR group (8.30; IQR, 7.40–9.30 g/dL) than those observed in the low PVR group (9.65; IQR, 8.70–11.4 g/dL) (*p* = 0.002). Furthermore, most patients in the high PVR group were in stage III according to the R-ISS classification (n = 15; 55.6%), whereas most patients in the low PVR group were in stage II (n = 66; 67.3%) (*p* < 0.001). No significant differences in the chemotherapy regimens were observed between the high and low PVR groups.

### 3.5. Survival Analysis 

**Figure 5:**
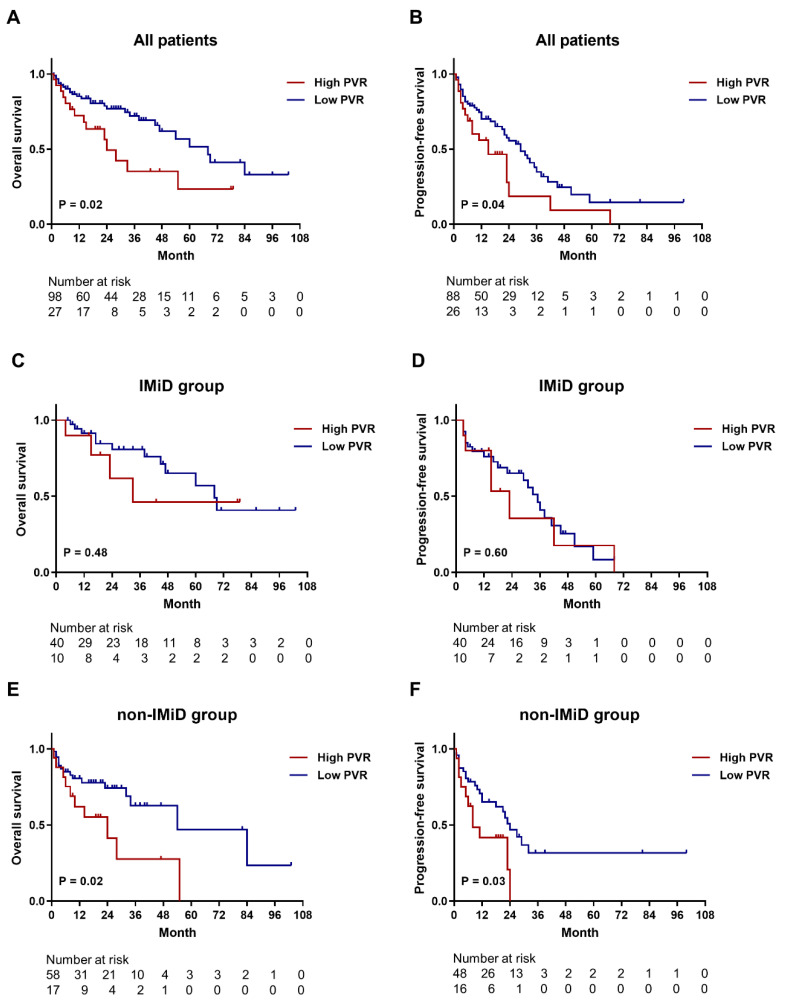
Kaplan–Meier survival curves for OS and PFS according to PVR expression. (**A**,**B**) OS and PFS curves for all patients. The median OS was 24 months in the high PVR-expression group and 68 months in the low PVR-expression group (n = 125). The median PFS was 15 and 29 months in the high and low PVR-expression groups, respectively (n = 114). (**C**,**D**) OS and PFS curves for the subgroups of patients (n = 50) who received IMiD therapy. The median OS (33 vs. 68 months) and PFS (23 vs. 35 months) showed no significant differences between low and high PVR-expression groups. (**E**,**F**) OS (n = 75) and PFS (n = 64) curves for the subgroups of patients who did not receive IMiD therapy. The median OS (24 vs. 54 months) and PFS (8 vs. 24 months) were significantly shorter in the low PVR-expression group relative to the high PVR-expression group. IMiD, immunomodulatory drug; OS, overall survival; PFS, progression-free survival; PVR, poliovirus receptor; TIGIT, T cell immunoreceptor with Ig and ITIM domains.

For investigating whether PVR expression is associated with the survival of patients with MM, we analyzed the OS and PFS of patients in relation to PVR-expression status. For PFS analysis, patients receiving only supportive treatment (n = 11) were excluded in accordance with the definition. Patient characteristics are summarized in Table S3. Among all patients, those with high expression of PVR showed significantly inferior OS compared to those with low expression (median, 24 vs. 68 months; *p* = 0.02) (Figure 5A), and those with high PVR expression also had significant inferior PFS compared to patients with low PVR expression (median, 15 vs. 29 months; *p* = 0.04) (Figure 5B). In the subgroup of patients who received immunomodulatory drug (IMiD)-based therapy, we observed no significant differences in OS (Figure 5C) or PFS (Figure 5D) between the two groups. Analysis of patients who received non-IMiD therapy revealed that those with high PVR expression had significantly shorter OS (median, 24 vs. 54 months; *p* = 0.02) (Figure 5E) and PFS (median, 8 vs. 24 months; *p* = 0.03) (Figure 5F) relative to those with low PVR expression. In addition, we conducted a Cox regression analysis of OS and PFS in relation to the best treatment response, which is well known as a major determinant of survival outcomes. Achievement of CR is a significant favorable prognostic factor for better OS (HR, 0.323; 95% CI, 0.127–0.823; *p* = 0.018) and PFS (HR, 0.499; 95% CI, 0.259–0.960; *p* = 0.037). 

### 3.6. Prognostic Implication of PVR Expression

For evaluating the prognostic effects of PVR expression, we used the Cox proportional hazard model (Table 3). In univariate analysis, the inferior prognosis was significantly correlated with an ECOG performance status ≥ 2, LDH levels > ULN, high-risk cytogenetics, and high PVR expression, whereas the favorable prognosis was significantly correlated with an achievement of complete response. Additionally, poor prognosis was associated with older age with borderline significance (*p* = 0.052). Multivariate analysis, using the backward stepwise elimination method that included all of the variables used in univariate analysis, showed that poor OS was independently predicted by high PVR expression [hazard ratio (HR), 2.029; 95% confidence interval (CI): 1.003–4.103; *p* = 0.048], ECOG performance status ≥ 2 (HR, 3.768; 95% CI: 1.185–11.983; *p* = 0.025), LDH levels > ULN (HR, 2.069; 95% CI: 1.040–4.113; *p* = 0.038), and high-risk cytogenetics (HR, 2.373; 95% CI: 1.165–4.843; *p* = 0.017). Collectively, these findings suggested PVR expression as a possible independent poor prognostic factor for survival in patients with MM.

## 4. Discussion

This study determined the prognostic value of PVR expression in patients with MM. We found that high PVR expression predicted inferior prognosis. Moreover, PVR expression was correlated with the presence of EMP, which indicates the presence of metastatic lesions, bone marrow plasma cells, and β2-microglobulin levels that are associated with tumor burden. Furthermore, multivariate analysis confirmed that high PVR expression was associated with poor OS in patients with MM.

The effect of PVR on the prognosis of cancer patients may be mediated by endogenous biological and immunological functions [1]. First, according to its endogenous function, overexpressed PVR supports proto-carcinogenic roles by promoting tumor cell invasion, migration, proliferation, and angiogenesis [1]. Moreover, PVR overexpression is associated with poor prognosis and enhanced tumor progression [1,8,9,10,12]. In this respect, we examined PVR expression in relation to the R-ISS stage and EMP, both of which were positively associated with PVR expression according to the H-score. Additionally, patients with elevated PVR expression showed a higher tumor burden based on the percentage of bone marrow plasma cells and β2-microglobulin, the levels of which are reportedly correlated with tumor stage [26], than those with lower PVR expression. Second, several recent studies focused on the immune effects of PVR protein [1]. For example, PVR overexpression in tumor cells increases the activation of immune cells and tumor cell death through interaction with CD226 [27]. In contrast, TIGIT and CD96 are inhibitory receptors, and PVR upregulation in heterogeneous tumor cells results in tumor cell recognition and binding by activating receptor CD226 and the inhibitory receptors TIGIT and CD96 [27]. A previous study noted that PVR shows the highest binding affinity for TIGIT, followed by CD96 and CD226, with these effects acting as immune checkpoints [2]. Thus, we examined TIGIT expression in bone marrow CD8^+^ T cells and NK cells and evaluated its association with PVR and clinical factors in patients with MM. In our study, we found that PVR expression did not correlate with TIGIT expression, although TIGIT expression in CD8^+^ T cells was positively associated with the percentage of bone marrow plasma cells but not β2-microglobulin levels or other clinical factors. However, we examined TIGIT expression in the 14 available samples and did not investigate functional interactions between PVR and TIGIT; therefore, further studies are required to clarify these results.

Previous studies have reported the prognostic implications of PVR expression in various solid and hematological cancer patients. Overall, these studies showed consistent results, indicating that elevated PVR expression is associated with decreased survival in patients with solid cancer, including lung cancer [8,28], esophageal cancer [9], cholangiocarcinoma [10], and breast cancer [12,29], and hematologic cancers, including AML [13]. However, the prognostic potential of PVR in patients with MM is still unclear. In the present study, survival outcomes of patients with MM were associated with PVR expression. Interestingly, subgroup analyses showed that PVR expression did not affect survival outcomes in patients treated with IMiD therapy. These findings contradict the independent correlation of PVR expression with survival outcomes in patients treated with non-IMiD therapy. Presumably, these findings suggest that IMiD modulates the immune effects of PVR in MM. For example, when PVR is highly expressed, immune evasion is predominant, and IMiD might enhance the antitumor response by involving the PVR-related immune pathway. Although IMiD therapy or combination IMiD therapy with a PVR inhibitor could be a promising strategy depending on PVR expression status, confirmatory conclusions on the relationship between PVR and IMiD therapy cannot be drawn from the present results, as we only observed differences in OS and PFS related to high PVR expression in patients who received IMiD therapy. Therefore, additional studies are required to identify the effects of IMiDs in relation to PVR expression in patients with MM. 

PVR is produced as a transmembrane protein with two alternate splicing isoforms (α and δ) that produce variants lacking transmembrane regions (designated as β and γ) [1,30], with PVRβ and PVRγ described as soluble and secreted isoforms, respectively [31]. Thus, we analyzed the soluble PVRβ isoform in the present study. A previous study demonstrated significantly higher levels of soluble PVR in patients with cancer relative to healthy donors, with these levels positively correlated with tumor stage [32]. Furthermore, high levels of soluble PVR are reportedly associated with poor prognosis in several cancer types, including hepatocellular carcinoma, breast cancer, and AML [33,34,35]; however, the role of soluble PVR in MM has not been studied. Therefore, this represents the first analysis of soluble PVR levels in patients with MM, as well as those with MGUS and without cancer. The results showed that patients with MM had higher soluble PVR levels than non-cancer patients, although no significant differences were observed between patients with MGUS and MM. We then analyzed the correlation between membrane and soluble PVR expression, revealing that soluble PVR levels correlated with membrane PVR levels detected by various methods. Although the potential differences between the two forms of PVR have not been studied in detail, measuring soluble PVR expression in bone marrow plasma cells may be a simpler and easier method for prognostication and treatment selection. Additionally, previous studies reported that TIGIT exists as a soluble protein [11,36], and the present results showed that soluble TIGIT levels in plasma correlated with its expression in bone marrow CD8^+^ T cells and NK cells. However, we observed no significant correlation between soluble PVR and TIGIT levels in patients with MM.

This study has several limitations. First, the most accurate methods for determining PVR expression in bone marrow specimens were not defined. Although double-staining IHC and H-score were used in this study, it is necessary to establish standardized methods. Second, an optimal cut-off value for high or low PVR expression measured with IHC has not been reported. Although we established a study-defined cut-off value based on our reported statistical method, a more standardized and reliable method is required. Third, we could not perform more in-depth molecular analyses; therefore, combined functional analysis of the effects of PVR overexpression would help clarify the mechanism related to the prognostic effects of PVR expression. Fourth, we did not investigate the expression of CD226, which is a co-stimulatory molecule opposed to TIGIT and influences TIGIT interactions with immune cells. This experiment will be conducted in a follow-up study. Furthermore, as we included patients with newly diagnosed MM, our findings may not be applicable to relapsed and refractory MM, making it necessary to conduct studies evaluating the prognostic value of PVR in these subsets of MM patients. Finally, the study mainly relied on retrospective analyses and included a relatively small number of patients. To reduce patient selection bias, we analyzed 22 sequentially obtained bone marrow aspiration (blood) samples using ELISA and qPCR to measure PVR expression; however, we could not test all bone marrow aspiration samples. Thus, confirmatory conclusions cannot be drawn from the results of this study.

In conclusion, we found that PVR expression was associated with poor prognosis in patients with newly diagnosed MM, suggesting its role as a potential prognostic marker. Additionally, the findings suggested that IMiD therapy may present a clinical benefit for patients having high PVR expression in MM. Follow-up studies are required to investigate the prognostic effects of the combination of PVR and its ligands, including CD226, CD96, and TIGIT, in MM.

## Figures and Tables

**Table 1:** Expression of PVR using different detection methods.

Patient	IHC (H-Score)	ELISA (ng/mL)	qPCR (Fold, *PVR*/*GAPDH*)
#1	2	0.251	0.520
#2	0	0.078 *	0.348
#3	4	0.311	2.815
#4	3	0.181	1.643
#5	0	0.173	2.362
#6	0	0.078 *	5.528
#7	2	0.243	0.143
#8	2	0.250	0.751
#9	2	0.276	1.170
#10	6	0.333	6.619
#11	6	0.166	1.248
#12	4	0.246	0.443
#13	2	0.167	0.475
#14	0	0.078 *	0.227
#15	2	0.218	0.109
#16	2	0.188	0.373
#17	2	0.199	0.729
#18	0	0.182	0.223
#19	0	0.237	0.199
#20	0	0.180	0.285
#21	2	0.166	0.061
#22	5	0.242	6.176

* Values were below the detection limit range and replaced with half of the limit of detection values for statistical analysis. ELISA, enzyme-linked immunosorbent assay; GAPDH, glyceraldehyde 3-phosphate dehydrogenase; IHC, immunohistochemistry; PVR, poliovirus receptor; qPCR, quantitative polymerase chain reaction.

**Table 2:** Comparison of clinical factors between patients with low and high PVR expression.

	Total (n = 125)	Low (n = 98)	High (n = 27)	*p*
Age, y	66.0 (58.0–72.0)	66.0 (58.0–72.3)	66.0 (58.0–72.0)	0.865
Sex, female	56 (44.8)	46 (46.9)	10 (37.0)	0.360
ECOG PS, ≥2	8 (6.4)	6 (6.1)	2 (7.4)	0.682
BM plasma cells, %	32.6 (15.6–61.3)	30.4 (12.8–51.9)	46.5 (25.9–77.0)	0.018
Serum M-protein, g/dL	2.20 (0.45–4.75)	2.12 (0.39–4.63)	3.30 (0.50–5.00)	0.350
Albumin, g/dL	3.30 (2.75–3.90)	3.40 (2.80–3.90)	2.90 (2.50–3.70)	0.088
<3.5 g/dL	71 (56.8)	54 (55.1)	17 (63.0)	0.465
β2-microglobulin, mg/L	4.87 (3.07–8.35)	4.44 (2.66–7.87)	6.63 (5.39–10.5)	0.004
≥5.5 mg/L	53 (42.4)	35 (35.7)	18 (66.7)	0.004
LDH, IU/L	390 (303–480)	363 (297–463)	434 (391–570)	0.070
>ULN	53 (42.4)	37 (37.8)	16 (59.3)	0.045
Calcium, mg/dL	9.10 (8.35–9.75)	9.20 (8.48–9.70)	8.50 (7.80–10.5)	0.886
>11 mg/dL	12 (9.6)	4 (6.7)	8 (12.3)	0.285
Creatinine, mg/dL	1.07 (0.81–1.84)	1.03 (0.80–1.53)	1.20 (1.01–2.21)	0.123
>2 mg/dL	24 (19.2)	10 (16.7)	14 (21.5)	0.490
Hb, g/dL	9.50 (8.30–11.3)	9.65 (8.70–11.4)	8.30 (7.40–9.30)	0.002
<10 g/dL	75 (60.0)	28 (46.7)	47 (72.3)	0.003
Cytogenetic abnormalities				
High risk *	41 (32.8)	28 (28.6)	13 (48.1)	0.055
ISS				
Stage I	26 (20.8)	25 (25.5)	1 (3.7)	0.006
Stage II	46 (36.8)	38 (38.8)	8 (29.6)	
Stage III	53 (42.4)	35 (35.7)	18 (66.7)	
R-ISS				
Stage I	14 (11.2)	14 (14.3)	0 (0.0)	<0.001
Stage II	78 (62.4)	66 (67.3)	12 (44.4)	
Stage III	33 (26.4)	18 (18.4)	15 (55.6)	
CTx regimen, 1st				
VTD	26 (20.8)	22 (22.4)	4 (14.8)	0.381
TD or RD	24 (19.2)	17 (17.3)	7 (25.9)	
VMP	57 (45.6)	45 (45.9)	12 (44.4)	
Others	7 (5.6)	4 (4.1)	3 (11.1)	
Supportive only	11 (8.8)	10 (10.2)	1 (3.7)	
CTx regimen, 2nd (n = 62)				0.864 †
KRD or IRD	22 (35.5)	20 (43.5)	2 (12.5)	
TD or RD	15 (24.2)	8 (17.4)	7 (43.8)	
VD	21 (33.9)	16 (34.8)	5 (31.2)	
Others	4 (6.5)	2 (4.3)	2 (12.5)	
CTx regimen, 3rd (n = 32)				0.296 †
KRD or IRD	7 (21.9)	4 (16.7)	3 (37.5)	
RD or PD	19 (59.4)	14 (58.3)	5 (62.5)	
VD	4 (12.5)	4 (16.7)	0	
Others	2 (6.2)	2 (8.3)	0	
Transplantation				
Auto-SCT	30 (24.0)	22 (22.4)	8 (29.6)	0.439
Allo-SCT	0	0	0	
None	95 (76.0)	76 (77.6)	19 (70.4)	

Values are number (percentage) or median (interquartile range). * High-risk cytogenetics: t(4;14), t(14;16), del(17/17p), *TP53* deletion, or chromosome 1 abnormalities [gain(1q) and del(1p)]. † Statistical differences were calculated between the immunomodulatory imide drug (IMiD) (KRD, IRD, TD, RD, and PD) and non-IMiD (VD and others) groups. BM, bone marrow; CTx, chemotherapy; ECOG, Eastern Cooperative Oncology Group; IRD, ixazomib, lenalidomide, and dexamethasone; ISS, International Staging System; Hb, hemoglobin; KRD, carfilzomib, lenalidomide, and dexamethasone; LDH, lactate dehydrogenase; PD, pomalidomide and dexamethasone; PS, performance status; PVR, poliovirus receptor; RD, lenalidomide and dexamethasone; R-ISS, Revised International Staging System; SCT, stem cell transplantation; TD, thalidomide and dexamethasone; ULN, upper limit of normal; VD, bortezomib and dexamethasone; VMP, bortezomib, melphalan, and prednisone; VTD, bortezomib, thalidomide, and dexamethasone.

**Table 3:** Univariate and multivariate analyses for OS.

Prognostic Factors	Univariate			Multivariate		
	HR	95% CI	*p*	HR	95% CI	*p*
Age, y	1.032	1.000, 1.065	0.052	1.032	0.997, 1.068	0.075
ECOG PS, ≥2	3.142	1.097, 8.994	0.033	3.768	1.185, 11.983	0.025
BM plasma cells, %	1.004	0.992, 1.016	0.525			
Serum M-protein, mg/dL	0.950	0.835, 1.080	0.434	0.887	0.765, 1.028	0.111
Albumin, g/dL	0.900	0.595, 1.359	0.615			
β2-microglobulin, mg/L	1.041	0.987, 1.099	0.139			
LDH, >ULN	2.912	1.545, 5.488	0.001	2.069	1.040, 4.113	0.038
Cytogenetics, high-risk *	2.072	1.086, 3.956	0.027	2.373	1.165, 4.834	0.017
Achievement of CR	0.323	0.127, 0.823	0.018			
PVR expression, high	2.127	1.114, 4.065	0.022	2.029	1.003, 4.103	0.048

* High-risk cytogenetics were defined as t(4;14), t(14;16), del(17/17p), *TP53* deletion, or chromosome 1 abnormalities, including gain(1q) and del(1p). BM, bone marrow; CI, confidence interval; CR, complete response; ECOG, Eastern Cooperative Oncology Group; HR, hazard ratio; LDH, lactate dehydrogenase; OS, overall survival; PS, performance status; PVR, poliovirus receptor; ULN, upper limit of normal.

## Data Availability

The datasets generated and/or analyzed during the current study are available from the corresponding author upon reasonable request.

## Supplementary Materials

The following supporting information can be downloaded at: https://www.mdpi.com/article/10.3390/biomedicines10051099/s1, Table S1: Original data for TIGIT expression by flow cytometry; Table S2: ELISA data for soluble PVR and TIGIT protein levels; Table S3: Baseline characteristics of 125 patients with multiple myeloma; Figure S1: Comparison of bone remodeling markers based on PVR expression status.

## References

[1] Kučan Brlić P., Lenac Roviš T., Cinamon G., Tsukerman P., Mandelboim O., Jonjić S. (2019). Targeting PVR (CD155) and its receptors in anti-tumor therapy. Cell. Mol. Immunol..

[2] Liu L., You X., Han S., Sun Y., Zhang J., Zhang Y. (2021). CD155/TIGIT, a novel immune checkpoint in human cancers (review). Oncol. Rep..

[3] Bronte V. (2018). The expanding constellation of immune checkpoints: A DNAMic control by CD155. J. Clin. Investig..

[4] Takai Y., Miyoshi J., Ikeda W., Ogita H. (2008). Nectins and nectin-like molecules: Roles in contact inhibition of cell movement and proliferation. Nat. Rev. Mol. Cell Biol..

[5] Smazynski J., Hamilton P.T., Thornton S., Milne K., Wouters M.C.A., Webb J.R., Nelson B.H. (2020). The immune suppressive factors CD155 and PD-L1 show contrasting expression patterns and immune correlates in ovarian and other cancers. Gynecol. Oncol..

[6] Masson D., Jarry A., Baury B., Blanchardie P., Laboisse C., Lustenberger P., Denis M.G. (2001). Overexpression of the CD155 gene in human colorectal carcinoma. Gut.

[7] Duan X., Liu J., Cui J., Ma B., Zhou Q., Yang X., Lu Z., Du Y., Su C. (2019). Expression of TIGIT/CD155 and correlations with clinical pathological features in human hepatocellular carcinoma. Mol. Med. Rep..

[8] Sun Y., Luo J., Chen Y., Cui J., Lei Y., Cui Y., Jiang N., Jiang W., Chen L., Chen Y. (2020). Combined evaluation of the expression status of CD155 and TIGIT plays an important role in the prognosis of LUAD (lung adenocarcinoma). Int. Immunopharmacol..

[9] Zhao K., Ma L., Feng L., Huang Z., Meng X., Yu J. (2020). CD155 overexpression correlates with poor prognosis in primary small cell carcinoma of the esophagus. Front. Mol. Biosci..

[10] Huang D.W., Huang M., Lin X.S., Huang Q. (2017). CD155 expression and its correlation with clinicopathologic characteristics, angiogenesis, and prognosis in human cholangiocarcinoma. OncoTargets Ther..

[11] Lozano E., Mena M.P., Díaz T., Martin-Antonio B., León S., Rodríguez-Lobato L.G., Oliver-Caldés A., Cibeira M.T., Bladé J., Prat A. (2020). Nectin-2 expression on malignant plasma cells is associated with better response to TIGIT blockade in multiple myeloma. Clin. Cancer Res..

[12] Stamm H., Oliveira-Ferrer L., Grossjohann E.M., Muschhammer J., Thaden V., Brauneck F., Kischel R., Müller V., Bokemeyer C., Fiedler W. (2019). Targeting the TIGIT-PVR immune checkpoint axis as novel therapeutic option in breast cancer. Oncoimmunology.

[13] Stamm H., Klingler F., Grossjohann E.M., Muschhammer J., Vettorazzi E., Heuser M., Mock U., Thol F., Vohwinkel G., Latuske E. (2018). Immune checkpoints PVR and PVRL2 are prognostic markers in AML and their blockade represents a new therapeutic option. Oncogene.

[14] Harjunpää H., Guillerey C. (2020). TIGIT as an emerging immune checkpoint. Clin. Exp. Immunol..

[15] Yeo J., Ko M., Lee D.H., Park Y., Jin H.S. (2021). TIGIT/CD226 axis regulates anti-tumor immunity. Pharmaceuticals.

[16] Liang R., Zhu X., Lan T., Ding D., Zheng Z., Chen T., Huang Y., Liu J., Yang X., Shao J. (2021). TIGIT promotes CD8^+^ T cells exhaustion and predicts poor prognosis of colorectal cancer. Cancer Immunol. Immunother..

[17] Tang W., Pan X., Han D., Rong D., Zhang M., Yang L., Ying J., Guan H., Chen Z., Wang X. (2019). Clinical significance of CD8^+^ T cell immunoreceptor with ig and ITIM domains+ in locally advanced gastric cancer treated with SOX regimen after D2 gastrectomy. Oncoimmunology.

[18] Liu X., Li M., Wang X., Dang Z., Jiang Y., Wang X., Kong Y., Yang Z. (2019). PD-1^+^ TIGIT^+^ CD8^+^ T cells are associated with pathogenesis and progression of patients with hepatitis B virus-related hepatocellular carcinoma. Cancer Immunol. Immunother..

[19] Xiao K., Xiao K., Li K., Xue P., Zhu S. (2021). Prognostic role of TIGIT expression in patients with solid tumors: A meta-analysis. J. Immunol. Res..

[20] Yang Z.Z., Kim H.J., Wu H., Jalali S., Tang X., Krull J.E., Ding W., Novak A.J., Ansell S.M. (2020). TIGIT expression is associated with T-cell suppression and exhaustion and predicts clinical outcome and anti-PD-1 response in follicular lymphoma. Clin. Cancer Res..

[21] Kong Y., Zhu L., Schell T.D., Zhang J., Claxton D.F., Ehmann W.C., Rybka W.B., George M.R., Zeng H., Zheng H. (2016). T-cell immunoglobulin and ITIM domain (TIGIT) associates with CD8^+^ T-cell exhaustion and poor clinical outcome in AML patients. Clin. Cancer Res..

[22] Liu G., Zhang Q., Yang J., Li X., Xian L., Li W., Lin T., Cheng J., Lin Q., Xu X. (2022). Increased TIGIT expressing NK cells with dysfunctional phenotype in AML patients correlated with poor prognosis. Cancer Immunol. Immunother..

[23] Guillerey C., Harjunpää H., Carrié N., Kassem S., Teo T., Miles K., Krumeich S., Weulersse M., Cuisinier M., Stannard K. (2018). TIGIT immune checkpoint blockade restores CD8^+^ T-cell immunity against multiple myeloma. Blood.

[24] Rajkumar S.V. (2016). Updated diagnostic criteria and staging system for multiple myeloma. Am. Soc. Clin. Oncol. Educ. Book.

[25] Contal C., O’Quigley J. (1999). An application of changepoint methods in studying the effect of age on survival in breast cancer. Comp. Stat. Data Anal..

[26] D’Anastasi M., Notohamiprodjo M., Schmidt G.P., Dürr H.R., Reiser M.F., Baur-Melnyk A. (2014). Tumor load in patients with multiple myeloma: Beta2-microglobulin levels versus whole-body MRI. AJR Am. J. Roentgenol..

[27] Molfetta R., Zitti B., Lecce M., Milito N.D., Stabile H., Fionda C., Cippitelli M., Gismondi A., Santoni A., Paolini R. (2020). CD155: A multi-functional molecule in tumor progression. Int. J. Mol. Sci..

[28] You H., Zhang Y.Z., Lai H.L., Li D., Liu Y.Q., Li R.Z., Khan I., Hsiao W.W., Duan F.G., Fan X.X. (2020). Prognostic significance of tumor poliovirus receptor and CTLA4 expression in patients with surgically resected non-small-cell lung cancer. J. Cancer Res. Clin. Oncol..

[29] Yong H., Cheng R., Li X., Gao G., Jiang X., Cheng H., Zhou X., Zhao W. (2019). CD155 expression and its prognostic value in postoperative patients with breast cancer. Biomed. Pharmacother..

[30] Koike S., Horie H., Ise I., Okitsu A., Yoshida M., Iizuka N., Takeuchi K., Takegami T., Nomoto A. (1990). The poliovirus receptor protein is produced both as membrane-bound and secreted forms. EMBO J..

[31] Baury B., Masson D., McDermott B.M., Jarry A., Blottière H.M., Blanchardie P., Laboisse C.L., Lustenberger P., Racaniello V.R., Denis M.G. (2003). Identification of secreted CD155 isoforms. Biochem. Biophys. Res. Commun..

[32] Iguchi-Manaka A., Okumura G., Kojima H., Cho Y., Hirochika R., Bando H., Sato T., Yoshikawa H., Hara H., Shibuya A. (2016). Increased soluble CD155 in the serum of cancer patients. PLoS ONE.

[33] Jin A.L., Yang Y.H., Su X., Yang W.J., Liu T., Chen W., Li T., Ding L., Wang H., Wang B.L. (2022). High serum soluble CD155 level predicts poor prognosis and correlates with an immunosuppressive tumor microenvironment in hepatocellular carcinoma. J. Clin. Lab. Anal..

[34] Iguchi-Manaka A., Okumura G., Ichioka E., Kiyomatsu H., Ikeda T., Bando H., Shibuya A., Shibuya K. (2020). High expression of soluble CD155 in estrogen receptor-negative breast cancer. Breast Cancer.

[35] Azzazi M., Hegab H., Abdelallah N.E.L.H., Mohamed H. (2020). AML-126: Prognostic value of serum CD 155 in adult acute myeloid leukemia patients: Relation to clinical outcome. Clin. Lymphoma Myeloma Leuk..

[36] Lozano E., Dominguez-Villar M., Kuchroo V., Hafler D.A. (2012). The TIGIT/CD226 axis regulates human T cell function. J. Immunol..

