# Supplementary material for: PVR (CD155) Expression as a Potential Prognostic Marker in Multiple Myeloma
Source: Biomedicines. 2022 May 10;10(5):1099. doi: 10.3390/biomedicines10051099 (PMC9139015; doi:10.3390/biomedicines10051099)
Supplement: Supplementary file 1 [file biomedicines-10-01099-s001.zip › biomedicines-1669122-supplementary.pdf]

Table S1. Original data for TIGIT expression by flow cytometry

Patient	$CD8^+$ T cells, %	NK cells, %
#1	4.53	14.1
#2	4.72	13.4
#3	2.28	1.68
#4	2.15	6.94
#5	9.16	69.7
#6	5.24	15.4
#7	2.62	12.1
#8	5.29	10.4
#9	0.14	21.1
#10	1.75	2.25
#11	2.93	23.5
#12	1.45	8.90
#13	7.12	50.9
#14	1.74	8.83

Table S2. ELISA data for soluble PVR and TIGIT protein levels

Patient	Soluble PVR, ng/mL	Soluble TIGIT, ng/mL
#1	1.908	3.056
#2	0.798	3.677
#3	6.918	5.425
#4	3.126	2.846
#5	1.225	16.370
#6	2.581	5.003
#7	1.633	3.347
#8	3.094	3.554
#9	2.415	2.831
#10	3.000	3.973
#11	2.158	5.499
#12	1.607	2.897
#13	0.320	19.290
#14	0.092	4.236
#15	0.085	22.151
#16	< 0.050	4.612
#17	0.522	3.003
#18	2.383	3.051
#19	2.685	2.653
#20	< 0.050	2.657
#21	1.424	4.161
#22	4.973	3.111

Table S3. Baseline characteristics of 125 patients with multiple myeloma

	Total (n = 125)	Non-IMiD (n = 75)	IMiD (n = 50)
Age, y	66.0 (58.0–72.0)	71.0 (66.0–76.0)	57.5 (52.0–62.0)
Sex, female	56 (44.8)	31 (41.3)	25 (50.0)
ECOG performance status, ≥ 2	8 (6.4)	5 (6.7)	3 (6.0)
BM plasma cells, %	32.6 (15.6–61.3)	32.9 (15.3–64.4)	28.0 (15.1–55.3)
Serum M-protein	2.20 (0.45–4.75)	3.40 (0.90–4.90)	1.30 (0.20–4.00)
Albumin	3.30 (2.75–3.90)	3.20 (2.70–3.70)	3.60 (2.90–4.15)
< 3.5 g/dL	71 (56.8)	50 (66.7)	21 (42.0)
β2-microglobulin	4.87 (3.07–8.35)	5.43 (3.14–9.50)	4.52 (2.48–7.84)
≥ 5.5 mg/L	53 (42.4)	35 (46.7)	18 (36.0)
LDH	390 (303–480)	393 (303–485)	372 (299–480)
≥ ULN	53 (42.4)	32 (42.7)	21 (42.0)
Calcium	9.10 (8.35–9.75)	8.90 (8.30–9.60)	9.25 (8.40–9.93)
> 11 mg/dL	12 (9.6)	7 (9.3)	5 (10.0)
Creatinine	1.07 (0.81–1.84)	1.15 (0.86–1.83)	1.01 (0.80–1.86)
> 2 mg/dL	24 (19.2)	15 (20.0)	9 (18.0)
Hb	9.50 (8.30–11.3)	9.20 (8.20–10.4)	10.0 (8.68–12.2)
< 10 g/dL	75 (60.0)	50 (66.7)	25 (50.0)
Bone lesion			
≥ 1 lesion	106 (84.8)	63 (84.0)	43 (86.0)
Cytogenetic abnormalities			
High-risk*	41 (32.8)	24 (32.0)	17 (34.0)
ISS			
Stage I	26 (20.8)	11 (14.7)	15 (30.0)
Stage II	46 (36.8)	29 (38.7)	17 (34.0)
Stage III	53 (42.4)	35 (46.7)	18 (36.0)
R-ISS			
Stage I	14 (11.2)	6 (8.0)	8 (16.0)
Stage II	78 (62.4)	44 (58.7)	34 (68.0)
Stage III	33 (26.4)	25 (33.3)	8 (16.0)

Values are number (percentage) or median (interquartile range). * High-risk cytogenetics were defined as t(4;14), t(14;16), del(17/17p), *TP53* deletion, or chromosome 1 abnormalities including gain(1q) and del(1p). BM, bone marrow; ECOG, Eastern Cooperative Oncology Group; IMiD, immunomodulatory drug; ISS, International Staging System; Hb, hemoglobin; LDH, lactate dehydrogenase; R-ISS, Revised International Staging System; ULN, upper limit of normal.

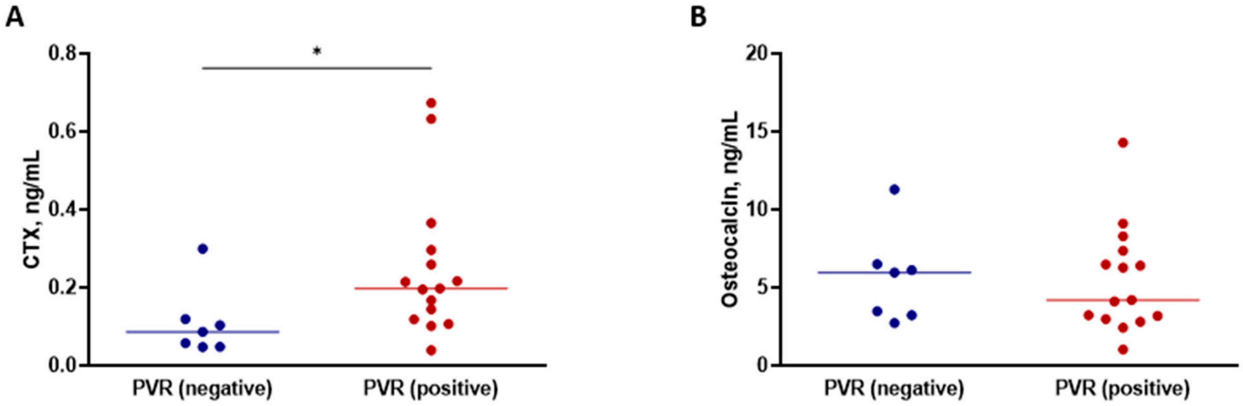

Figure S1. Comparison of bone remodeling markers based on PVR expression status. **(a)** C-telopeptide of type I collagen (CTX), a bone resorption marker and **(b)** osteocalcin, a bone formation marker. * $p < 0.05$; Mann–Whitney *U* tests.
